# Factors Associated with Relapse among Heroin Addicts: Evidence from a Two-Year Community-Based Follow-Up Study in China

**DOI:** 10.3390/ijerph13020177

**Published:** 2016-01-28

**Authors:** Chao Rong, Hai-Feng Jiang, Rui-Wen Zhang, Li-Juan Zhang, Jian-Chen Zhang, Jing Zhang, Xue-Shan Feng

**Affiliations:** 1 School of Public Health, Fudan University, Shanghai 200032, China; xsfeng@shmu.edu.cn; 2 School of Humanities and Social Sciences, Zhejiang Chinese Medical University, Hangzhou 310053, China; 3 Shanghai Mental Health Center, Shanghai 200030, China; rcnj2008@163.com; 4 Technological and Industrial Promotion Center of Traditional Chinese Medicine, Shanghai 201203, China; guanlishui@126.com (R.-W.Z.); zhanglj@biopham.sh.cn (L.-J.Z.); 5 Drug Control Office, Shanghai 200129, China; zhangjc88@sina.com; 6 The Council of Shanghai Ziqiang Social Services, Shanghai 200030, China; isurevictory@163.com; 7 Key Laboratory of Public Health Security, Ministry of Education, Shanghai 200032, China

**Keywords:** community-based drug treatment, methadone, Jitai tablets, psychological counseling, follow-up study

## Abstract

*Background:* Many countries including China are facing a serious opiate dependence problem. Anti-drug work effectiveness was affected by the high relapse rate all over the world. This study aims to analyze the factors influencing heroin addict relapse, and to provide evidence for generating relapse prevention strategies. *Methods:* A community-based follow-up study was conducted in China between October 2010 and September 2012. A total of 554 heroin addicts in accordance with the inclusion criteria from 81 streets in 12 districts of Shanghai, China were divided into 4 groups: group 1—daily dosage taken orally of 60 mL of methadone or under combined with psychological counseling and social supports (*n* = 130); group 2—daily dosage taken orally of over 60 mL of methadone combined with psychological counseling and social supports (*n* = 50); group 3—JTT (Jitai tablets) combined with psychological counseling and social supports (*n* = 206); group 4—JTT combined with social supports (*n* = 168). *Results:* Log-rank test results showed that the cumulative relapse rate differences among four groups during the two-year follow-up period were not statistically significant (χ^2^ = 5.889, *p* = 0.117). Multivariate Cox regression analysis results showed that only three independent variables were still statistically significant, including compliance with participation in psychological counseling (OR = 3.563, *p* = 0.000), the years of drug use (OR = 1.078, *p* = 0.001)and intervention model. *Conclusions:* Using the detoxification medications combined with appropriate psychological counseling and social support measures will help improve the effectiveness of relapse prevention, which is a kind of alternative community detoxification pattern. Appropriate and standard psychological counseling is very important for anti-drug treatment. The longer the drug addiction lasts, the longer the anti-drug treatment takes.

## 1. Introduction

Many countries are facing a serious problem with opiate dependence. According to a report by the United Nations Office on Drugs and Crime (UNODC) from the Chinese Ministry of Public Security in 2014, global drug trafficking has involved more than 170 countries and regions with an annual drug trade volume over $800 billion, equivalent to 13% of the total amount of world trade. The number of global drug addicts is now 300 million. What’s more, each year more than 200,000 people are killed and 10 million people lose the ability to work as a result of drug use [1]. Opium, morphine and heroin and other opioid drugs cause the heaviest drug-related burden of disease and the most drug-related deaths in the world. China is also facing this challenge. At the end of April 2014, there were 2.58 million drug addicts registered in China, including 1.38 million opioid drug addicts, which make up 53%. According to the proportion of explicit and implicit drug addicts, the actual number of drug addicts in China is probably more than 10 million [2].

Opioid addiction is a severe public health problem which has severe consequences. It is both a social problem and a public health problem in that it not only adds to the economic burden of society and the family, but also causes serious damage to physical and mental health, resulting in loss of labor, broken families, participation in criminal activities, destruction of social harmony, the spread of AIDS, *etc.* [3]. The age of first drug use is decreasing, while larger dosages re becoming more popular with more types of drugs being used. At the same time, intravenous drug use is becoming more and more popular. Therefore, these drugs are enormously harmful to the national economy, the population and social stability [4].

Due to the great physical and mental dependency caused by opioid drugs, the vast majority of drug addicts relapse after detoxification treatment. For a long time, the high relapse rate has been affecting the effectiveness of anti-drug work all over the world. According to studies outside China, the relapse rate within 1st year is usually between 80% and 95% [5]. According to studies in China, after detoxification, the relapse rate for heroin abusers within the 1st month is 54.57%. The relapse rate within 1st to 3rd months is 31.76%, while it is 93.31% within the first 6 months, and 96.68% within 1st year. Drug addicts often fall into the vicious cycle of “drug-taking–detoxification–relapse–effort–quit”, constituting a worldwide problem in current anti-drug work [6].

Methadone maintenance treatment (MMT) program is defined as long-term (at least three months) use of methadone combined with psychological counseling and social support measures, in order to ultimately achieve reduction of drug demand and harm. The method requires that drug addicts take a daily dose of methadone under the supervision of staff at the designated place, thereby reducing the incidence of illicit drug use and related risk behaviors. MMT was first introduced to China in 2004. Initially, it was tried only in eight clinics in five provinces. Now it has been expanded to a nationwide program involving 680 clinics, which served 242,000 patients in 2009 [7]. MMT patients need to pay 10 yuan ($1.50) for their daily treatment and take methadone under the supervision of medical service personnel [8]. Numerous studies have shown that many patients continue to engage in drug-using behaviors during or after MMT program [9]. JTT has been approved for use in the treatment of drug addiction by China’s State Food and Drug Administration (SFDA).

So far, no optimal method has yet been found to eradicate drug addiction. A number of studies have shown that relapse is the result of interaction between many factors, which includes not only external environmental factors (natural environmental factors and social environmental factors), but also individual factors (genetic predisposition, personality characteristics, *etc.*) [10]. Existing studies have shown that JTT not only can effectively control withdrawal symptoms in heroin addicts but also can help relieve protracted withdrawal symptoms without obvious adverse effect and dependence [11]. However, currently there are no adequate quantitative studies of the link between relapse and some specific factors [12]. As a quantitative study, this study aims to analyze the correlations between relapse and some specific factors, in order to explain the relapse prevention effect and provide some basis for government and relevant functional departments to formulate intervention strategies for relapse prevention.

## 2. Material and Methods

### 2.1. Design, Setting and Participants

This study was based on a community-based cohort study designed for opioid addict intervention. The 554 subjects came from 81 streets and 12 districts in Shanghai (Huangpu, Putuo, Hongkou, Jingan, Xuhui, Yangpu, Zhabei, Baoshan, Changning, Minhang, Chongming and Pudong). Listed below are the inclusion and exclusion criteria.

Inclusion criteria (meeting all of them): (1) meet DSM-IV criteria for opioid dependence; (2) have completed acute detoxification treatment; (3) aged 18 to 65 years old; (4) subject to administration of police and anti-drug social workers; (5) adhere to medication and complete record of the relevant information; (6) informed consent by subjects or their legal guardians.

Exclusion criteria (meeting any one of the following conditions): (1) glaucoma disease or severe mental illness, or a history of epilepsy (except febrile convulsion in children); (2) serious organic disease in the past three months, such as gastrointestinal bleeding, *etc.*; (3) any one of the following: under other medication treatment, under other auxiliary treatment such as electroacupuncture for pain, or before 5th half-life of rehabilitation medicine and psychotropic drugs that have been used before inclusion (except the drugs allowed for combined use); (4) pregnant or breast-feeding women; (5) unwillingness to comply with the trial requirements; (6) obvious tendency toward impulse or impulse to commit suicide or self-injury; (7) abnormal ECG examination or abnormal laboratory results with obvious clinical significance considered by researchers that could affect efficacy and safety evaluation; (8) Alanine Transaminase (ALT) or Aspartate Transaminase (AST) five times higher than the normal maximum; (9) serious drug allergy history or known allergy to JTT.

This was an open cohort study of repeated measures and parallel control. The number of cases for recruitment was 612. A total of 554 subjects in accordance with the criteria were included in the study, while 157 of them were lost during follow-up. In total there were 407 subjects participating in the entire 2-year study. We found the reasons for loss included relapse, pregnancy, death, abandoning treatment, and robbery and other offenses. If the cause of loss was relapse, then it counted toward the number of relapses. According to the principle of informed and voluntary consent, 554 opioid addicts who had completion the acute detoxification in compulsory detoxification were assigned to different groups. Group 1 (130 cases) was administered a daily oral dosage 60 mL of methadone or under, combined with psychological counseling and social support measures, and group 2 (50 cases) was administered a daily oral dosage of over 60 mL of methadone combined with psychological counseling and social support measures, while group 3 (206 cases) was administered JTT combined with psychological counseling and social support measures, and group 4 (168 cases) was administered JTT combined with social support measures. If the MMT program was chosen, assignment to group 1 or group 2 depending on the physician’s professional judgment. A one-year intervention period and one-year observation period were given to all the four groups, including baseline screening and six follow-ups on the 8th, 26th, 52th, 64th, 78th, and 104th weeks.

### 2.2. Interventions

#### 2.2.1. Medication Methods

Methadone is an oral liquid whose dosage can vary according to subjects’ specific conditions, as decided by doctors, between 15 mL to 120 mL everyday [13]. JTT is non-narcotic detoxification-dedicated pure herbal medicine, which is based on the detoxification series recipe of one of the four famous TCM doctors (Li Shijian, Pang Anshi, Wan Mizhai, Yang Jitai) of eastern Hubei in the Ming and Qing Dynasties, Yang Jitai. JTT was derived from the essence of traditional Chinese medicine, with the use of modern science and technology and the latest process for modern drug characteristics, combined with clinical experience and animal studies and the international forefront of scientific innovation. The specification of JTT is 0.4 g/tablet which consists of 15 ingredients, including Ligusticum Chuanxiong, Radix angelicae sinensis, Radix salviae miltiorrhizae, Rhizoma corydalis, and Flos Daturae. JTT is administered orally under the supervision of family members and can be taken home. The patients received the supply of the tablets according to a community doctor’s prescription, and the remaining tablets were returned to the doctor at the next follow-up. The dosages for JTT were as follows: 3 tablets twice a day for 1st–8th weeks, 2 tablets twice a day for 9th–26th weeks, and then 1 tablet once a day for 27th–52th weeks, all taken after meals.

#### 2.2.2. Psychological Counseling

Psychological counseling included group and individual psychological counseling, mainly the former. Community-based group psychological counseling was implemented by social workers trained by counselors of Shanghai Mental Health Center with 8 to 10 people in a group. It was carried out once every two weeks, consecutively, 12 times in the first six months. There was a theme each time, lasting 1.5 h, including group discussions, cognitive therapy, scene training, improving the subjects’ self-related behaviors, rectifying the misunderstanding of families and society, and providing help and solutions in case of emergency, *etc.* Individual psychological counseling was carried out by the local counseling telephone hotline, which equals seeking psychological support in an active way.

#### 2.2.3. Social Support Measures

Social support measures included providing subsistence allowance, recommending employment, providing vocational training and medical expense reduction, reducing medical costs, providing home instruction, helping their children in their studies and so on. Social workers performed or assisted relevant community functional departments to help enforce these measures.

### 2.3. Relapse Definition

So far, there is still no generally accepted definition of relapse at home or abroad. In this study, relapse was defined as a urine test positive for morphine, or drug addicts arrested by public security departments to be compulsorily isolated due to relapse in the follow-up period (8th week, 26th week, 52th week, 64th week, 78th week and 104th week) [14].

### 2.4. Data Sources and Variable Assignment

The data was obtained from two sources. The first source was trained social workers through face-to-face interviews by using questionnaires, collecting information on age, gender ((1) male; (2) female), marital status ((1) married; (2) divorced; (3) unmarried; (4) other. The variable was set to dummy variable, and the group of the married people as reference group), educational level (1. primary school; 2. junior school; 3. high school; 4. college and above. The variable was set to dummy variable, and the group of the primary school people as reference group), occupational status in the last three years ((1) full-time; (2) part-time; (3) unemployed; (4) constrained environment; (5) students or others. The variable was set to dummy variable, and the group of the full-time people as reference group), the age of first drug use, years of drug use, previous detoxification number, the years of smoking , the amount of smoking, intervention model ((1) group 1; (2) group 2; (3) group 3; (4) group 4. The variable was set to dummy variable, and the group 1 as reference group). The second source was the follow-up CRF records and tests including urine drug test ((0) negative; (1) positive) and adverse events (e.g., an object was captured by the public security department because of relapse). The follow-up CRF records, tests and adverse events helps to determine whether there was relapse (Relapse is the dependent variable for the Cox regression analysis in this paper, 0. not relapse, 1. relapse). If the patient had not done a urine morphine test in a follow-up, then the result was reckoned according to the next follow-up test result. If the patient had not done any urine morphine tests in all the subsequent follow-ups, and they were not captured by the public security department for relapses, then the subjects were considered to be lost in follow-ups. The subjects were accompanied by social workers to do urine tests for morphine, and the social workers collected the test results. In addition, there was compliance with participating in psychological counseling (the actual number participating in psychological counseling divides the theoretical number of participating in psychological counseling×100%. (1) over 75% as good; (2) 50%–75% as moderate; (3) below 50% as poor. Group 1 was the reference group), and the Self-Rating Anxiety Scale (SAS, which is used to measure the severity of anxiety, with higher scores indicating more severe anxiety), Self-rating Depression Scale (SDS, which is used to measure the severity of depression, with higher scores indicating more severe depression), Visual Analog Scale (VAS, which is used to measure the severity of psychological craving and pain, with higher scores indicating more severe psychological craving and pain.) to measure the desire and pain, protracted withdrawal symptom rating scales, Addiction Severity Index (ASI, which is mainly used to assess the severity of the addiction. It determines the demand for treatment and can evaluate the treatment effect. possible score, 0 to 1, with higher scores indicating more severe addiction) and health survey (SF-36, which is the world’s most widely used quality of life assessment tools, with higher scores indicating better health). We took the average value of all previous follow-up evaluation scores of each scale as the independent variables [15].

### 2.5. Statistical Analysis

Categorical variables were described with frequencies and percentages. The chi-square test method was used to compare such differences, and the log-rank test (sequential test) method was used to compare the differences in relapse rates of the two years between the four groups. Continuous variables were described with the mean and standard deviation, and Wilcoxon test was used to compare baseline characteristics between the four groups. Considering that there were many influencing factors, the sample size may be insufficient (554 cases). Therefore, in this study the Cox single factor regression analysis method was used to screen the independent variables which were statistically significant, then the Cox multiple factors regression analysis method was used for the above statistically significant variables and the intervention model variable which should be considered by professional judgment. [16]. The SAS version 9.1 software was used for statistical analysis. All statistical tests were two-sided, and *p* ≤ 0.05 was considered statistically significant.

### 2.6. Ethics Statement

This study was approved by the Ethics Committee of Shanghai Mental Health Center (No. 2009-14-R). Subjects were informed of the relevant issues in detail before the study and informed consents were signed.

## 3. Results

### 3.1. Demographic Statistics and Baseline Measures

There were no significant differences in gender, age, marital status, education level and age of first drug use among the four groups. However, there were significant differences in occupational status, years of drug use, years of smoking and previous detoxification number in the baseline (Table 1 and Table 2).

**Table 1:** Participant characteristics (1) % (*n*).

Characteristic	Group 1 (*n* = 130)	Group 2 (*n* = 50)	Group 3 (*n* = 206)	Group 4 (*n* = 168)	χ^2^	*p*
Gender					4.919	0.178
Male	80.0% (108)	84.0% (42)	87.4% (180)	79.8% (134)		
Female	20.0% (24)	16.0% (8)	12.6% (26)	20.2% (34)		
Marital status					12.495	0.187
Married	36.9% (48)	28.0% (14)	24.8% (51)	23.8% (40)		
Divorced	21.5% (28)	36.0% (18)	27.2% (56)	25.6% (43)		
Unmarried	36.2% (47)	30.0% (15)	42.7% (88)	42.3% (71)		
Others	5.4% (7)	6.0% (3)	5.3% (11)	8.3% (14)		
Educational level					8.318	0.502
Primary	3.8% (5)	10.0% (5)	2.4% (5)	4.2% (7)		
Junior high school	63.8% (83)	60.0% (30)	66.5% (137)	69.6% (117)		
Senior high school	30.8% (40)	28.0% (14)	28.6% (59)	25.0% (42)		
College degree or above	1.5% (2)	2.0% (1)	2.4% (5)	1.2% (2)		
Occupational status					59.759	0.000 *
Full-time job	19.2% (25)	10.0% (5)	12.6% (26)	15.5% (26)		
Part-time job	6.9% (9)	18.0% (9)	7.3% (15)	5.4% (9)		
Unemployed	3.1% (4)	0.0% (0)	42.7% (88)	49.4% (83)		
Free-limited environment	60.8% (79)	70.0% (35)	36.9% (76)	28.0% (47)		
Student or others	10.0% (13)	2.0% (1)	0.5% (1)	1.8% (3)		

Notes: Bold values are statistically significant at *p* ≤ 0.05; CI: Confidence Interval; * *p* < 0.05.

**Table 2:** Participant characteristics (2) Mean ± SD.

Characteristic	Group 1 (*n* = 130)	Group 2 (*n* = 50)	Group 3 (*n* = 206)	Group 4 (*n* = 168)	χ^2^	*p*
Age	41.61 ± 8.20	43.92 ± 6.98	40.98 ± 8.02	40.24 ± 8.65	7.730	0.052
Age of first drug use	29.73 ± 8.18	30.80 ± 7.05	30.00 ± 8.33	30.10 ± 8.18	1.035	0.793
Years of drug use	9.10 ± 4.37	10.73 ± 4.00	8.26 ± 4.83	7.74 ± 4.51	20.802	0.000 *
Years of smoking	14.41 ± 10.05	16.65 ± 8.46	14.55 ± 10.14	11.68 ± 10.36	13.140	0.004 *
Previous detoxification times	3.56 ± 1.79	3.26 ± 1.50	2.93 ± 1.48	2.71 ± 1.69	23.111	0.000 *

Notes: Bold values are statistically significant at *p* ≤ 0.05; CI: Confidence Interval; * *p* <0.05.

### 3.2. The Treatment Outcomes of the Four Groups

The accumulative relapse rate of group 1 increased from 3.1% in 8 weeks to 13.8% in 104 weeks; the accumulative relapse rate of group 2 increased from 8.0% in 8 weeks to 20.0% in 104 weeks; the accumulative relapse rate of group 3 increased from 3.9% in 8 weeks to 23.3% in 104 weeks; the accumulative relapse rate of group 4 increased from 1.2% in 8 weeks to 24.4% in 104 weeks. The accumulative relapse rate differences among four groups in the 64th week were statistically significant, and the accumulative relapse rate of group 1 was significantly lower than group 2 (χ^2^ = 3.904, *p* = 0.048) and group 3 (χ^2^ = 7.892, *p* = 0.005). Log-rank test results showed that the cumulative relapse rate differences among four groups during the two years follow-up period were not statistically significant (χ^2^ = 5.889, *p* = 0.117) (Table 3).

**Table 3:** The number of accumulative relapse and accumulative relapse rate of four groups in each follow-up period.

Follow-Up Period	Group 1 (*n* = 130)	Group 2 (*n* = 50)	Group 3 (*n* = 206)	Group 4 (*n* = 168)	χ^2^	*p*
Week 8	4 (3.1%)	4 (8.0%)	8 (3.9%)	2 (1.2%)	6.131	0.105
Week 26	5 (3.8%)	6 (12.0%)	20 (9.7%)	11 (6.5%)	5.569	0.135
Week 52	11 (8.5%)	9 (18.0%)	33 (16.0%)	25 (14.5%)	4.757	0.190
Week 64	12 (9.2%)	10 (20.0%)	43 (20.9%)	28 (16.7%)	8.148	0.043 *
Week 78	16 (12.3%)	10 (20.0%)	47 (22.8%)	36 (21.4%)	6.081	0.108
Week 104	18 (13.8%)	10 (20.0%)	48 (23.3%)	41 (24.4%)	5.843	0.120

### 3.3. Analysis of Factors Influcing Relapse Based on Univariate and Multivariate Cox Regression Analysis Method

The independent variables of univariate Cox regression analysis included: age, gender, occupational status in the last three years, educational level, marital status, age of first drug use, years of drug use, years of smoking, amount of smoking, previous detoxification times, intervention model, compliance of participating in psychological counseling, average scores of Addiction Severity Index (ASI), protracted withdrawal symptom rating scales, average level of pain, health survey (SF-36), average craving for drugs, Self-Rating Anxiety Scale (SAS), and Self-rating Depression Scale (SDS) of the heroin addicts. The results of the univariate Cox regression analysis (Table 4) showed that seven independent variables are statistically significant, which included the intervention model and the compliance of participating in psychological counseling (*p* = 0.000), the years of drug use (*p =* 0.024), Addiction Severity Index (ASI) (*p* = 0.011), the protracted withdrawal symptom rating scales (*p* = 0.026), the average level of pain (*p* = 0.006) and the average craving for drugs (*p* = 0.000). Then, the method of multivariate Cox regression analysis was used for analyzing above statistically significant variables. The results showed that only three independent variables were still statistically significant, which included the years of drug use (OR = 1.078, *p* = 0.001), the compliance of participating in psychological counseling (OR = 3.563, *p* = 0.000) and the intervention model. That is, keeping the levels of other factors fixed, a one-year increase in the years of drug use results in a 0.078 unit increase in the relapse risk, one-level increase in the compliance with participation in psychological counseling results in a 2.563 times increase in the relapse risk, and the relapse risk of group 4 is 1.661 times higher than group 1 (Table 5).

**Table 4:** The result of the univariate Cox regression analyses.

Independent Variable	β	*p*	OR	95% CI
Age	−0.007	0.576	0.993	0.970–1.017
Gender	0.361	0.225	1.435	0.801–2.570
Occupational status (in the last three years):				
Full-time job (reference group)		0.271		
Part-time job	−1.191	0.056	0.304	0.089–1.032
Unemployed	0.173	0.817	1.189	0.276–5.123
Free-limited environment	−0.231	0.403	0.794	0.463–1.363
Student or others	0.040	0.896	1.040	0.575–1.881
Educational level:				
Primary (reference group)		0.606		
Junior high school	−0.073	0.888	0.930	0.338–2.557
Senior high school	0.220	0.678	1.246	0.441–3.516
College degree or above	−0.026	0.976	0.975	0.178–5.322
Marital status:				
Married (reference group)		0.421		
Divorced	0.013	0.961	1.013	0.610–1.681
Unmarried	−0.327	0.185	0.721	0.445–1.170
Others	−0.393	0.415	0.675	0.263–1.737
Intervention model:				
group 1 (reference group)		0.126		
group 2	0.593	0.121	1.810	0.855–3.832
group 3	0.373	0.196	1.452	0.825–2.558
group 4	0.669	0.021 *	1.952	1.105–3.449
Compliance of participating in psychological counseling	0.552	0.000 *	1.737	1.420–2.125
Age of first drug use	−0.013	0.298	0.987	0.963–1.012
Years of drug use	0.049	0.024 *	1.050	1.006–1.095
Previous detoxification number	−0.004	0.949	0.996	0.881–1.125
Years of smoking	0.008	0.423	1.008	0.988–1.029
Amount of smoking	0.019	0.118	1.019	0.995–1.044
addiction severity Index (ASI)	0.453	0.011 *	1.573	1.111–2.227
Protracted withdrawal symptom rating scales	0.020	0.026 *	1.021	1.002–1.039
Average level of pain	0.016	0.006 *	1.016	1.005–1.028
Physical health (SF-36)	−0.007	0.182	0.993	0.983–1.003
Mental health (SF-36)	−0.004	0.413	0.996	0.985–1.006
Average craving for drugs	0.023	0.000 *	1.023	1.013–1.033
Self-Rating Anxiety Scale (SAS)	0.017	0.080	1.017	0.998–1.037
Self-rating Depression Scale (SDS)	0.016	0.066	1.016	0.999–1.033

Notes: CI: Confidence Interval; * *p* <0.05; There are three kinds of ASI score, namely drug addict addiction self-rating estimate, severity of addiction estimated by the interviewer and dimension scoring. This study used the dimension scoring method.

**Table 5:** The result of the multivariate Cox regression analyses.

Independent Variable	β	*p*	OR	95% CI
Intervention model:				
Group 1 (reference group)		0.002		
Group 2	0.428	0.269	1.535	0.718–3.279
Group 3	0.027	0.929	1.028	0.565–1.868
Group 4	0.979	0.001 *	2.661	1.458–4.857
Compliance of participating in psychological counseling	1.271	0.000 *	3.563	2.611–4.862
Years of drug use	0.075	0.001 *	1.078	1.031–1.127
addiction severity Index (ASI)	0.014	0.947	1.014	0.668–1.541
Protracted withdrawal symptom rating scales	0.007	0.585	1.007	0.982–1.032
Average level of pain	0.007	0.373	1.007	0.991–1.024
Average craving for drugs	0.009	0.239	1.009	0.994–1.025

Notes: CI: Confidence Interval; * *p* <0.05; There are three kinds of ASI score, namely drug addict addiction self-rating estimate, severity of addiction estimated by the interviewer and dimension scoring. This study used the dimension scoring method.

## 4. Discussion

Drugs, especially opioid drugs, have strong physical and mental dependency properties, and the vast majority of addicts relapse after detoxification treatment. A number of studies have shown that drug relapse in patients was related to the three aspects of physical, psychological and social factors [17]. In methadone maintenance treatment areas, a previous study has shown that psychological counseling or other services can improve the effectiveness of methadone maintenance treatment. The opioid-dependent patients were divided into three groups in a well-known study: the minimum methadone services (MMS): only MMT (at least 60 mg daily) was provided; standard methadone services (SMS): MMT together with psychological counseling services; enhanced methadone services (EMS): MMT and psychological counseling services, together with medical, spiritual, family and employment treatment service. The intervention period was 24 weeks. The results showed that the positive proportion of MMS group urine testing for opioids was significantly higher than the other two groups, and the positive proportion of EMS group urine test of opioids was significantly lower than SMS. In all, the enhanced group therapy worked the best, followed by the standard group, while the minimum methadone group was the worst. This serves as a base in explaining the effects of psychological counseling and social support intervention besides drug treatment [15].

The mechanism of relapse still remains unclear. Moreover, relapse involves functional disorders of many parts of the nervous system, and thus cannot be antagonized by single-acting medication. While there is a series of problems caused by synthetic medicine, traditional anti-drug methods such as drug replacement therapy cannot effectively reduce psychological dependence. As a result, relapse rate remains high for a long time, making it very urgent to find new medications and comprehensive detoxification methods. In China detoxification research experts have turned to traditional Chinese medicine, hoping to be able to use multi-target regulation of Chinese medicine to solve the problem of relapse [18]. In this study, the JTT, an approved anti-drug Chinese medicine, does not contain any narcotic substance or any anesthetics. Shanghai Chinese Pharmaceutical Technology Co., Ltd. (Shanghai, China) was approved to produce JTT by China’s State Food and Drug Administration in 2004 (production approval number: Z20044197). China has rich resources of Chinese materia medica with relatively low prices. Modern Chinese medicine preparations can be taken orally, and is easy to use with comparatively few side effects. JTT shares the above advantages, and is not psychotropic medicine. Therefore, special control from government and medical institutions is not required (the use of methadone needs to follow the provisions of the “Measures for the management of narcotic drugs”), which makes it suitable for community detoxification for patients’ long-term use. It also conforms to the traditional folk medical culture [19]. The study results shows that relapse rates of the four groups are relatively low in the 2-year follow-up period, indicating using the detoxification medications combined with appropriate psychological counseling and social support measures can help to prevent relapse, making it a kind of alternative community detoxification pattern.

The multivariate analysis results of this study showed that the compliance of participating in psychological counseling and the intervention model was associated with relapse. Keeping the levels of other factors fixed, one-level decrease in the compliance of participating in psychological counseling results in a 2.563 times increase in the relapse risk (*p* = 0.000), the relapse risk of group 4 is 1.661 times higher than group 1. There are various factors affecting relapse, including psychological dependence on drugs, anxiety and depression in heroin addicts, morbid psychology like personality disorders and mood disorders. Compared with physiological dependence, psychological dependence is more tacit and lasting, and more difficult to withdraw. Relevant data showed that psychological dependence on drugs generally lasts 1 to 3 years, sometimes even a whole lifetime [11]. The study by Ma Jun *et al.* found that psychological addiction was the most important factor of relapse, accounting for 73.08% [20]. Some studies showed that drug addicts had more serious psychological and mental problems, mainly anxiety, depression, sensitivity, fear, anger, emotional instability and irritability. In addition, morbid psychology after drug taking easily leads to relapse. Gao Zhiqin *et al.* carried out the Minnesota Multiphasic Personality Inventory Survey for 109 heroin addicts who relapsed, and their hypochondria, depression, psychosis and paranoia scores were significantly higher than normal (*p* < 0.01) [21]. It indicates that such relapsing individuals have serious mental defects and personality problems, and they are susceptible to negative impact of life events which induce relapse. The study also showed that the group 1 and group 2 and group 3 intervention model for the effects of relapse is not significantly different; however, the group 1 and group 4 intervention model for the effects of relapse is significantly different. It explained that the difference in effect between MMT and JTT is not significant, and psychological counseling plays a significant role in prevention of relapse. Therefore, appropriate and standard psychological counseling is very important for drug treatment.

This study also found that years of drug use was associated with relapse (*p* = 0.001). Keeping levels of other factors fixed, one-level increase in the years of drug use results in a 0.078 unit increase in the relapse risk. The longer the years of drug addition, the bigger their neural biochemical mechanism changes, and the more unstable their psychological-behavioral adjustment modes are, and the stranger their living environment and lifestyle. Psychological causes are correlated with psychological reliance caused by central neurotransmitter changes. The longer the years of drug addition, the stronger the psychological dependence is, and more susceptible to depression, anxiety and other mental disorders. The longer the years of drug addiction, the more drug-using peers they will have. Time deepens these relationships, making the temptation and pressure from friends greater during drug treatment. Any psychological reason has its foundation in physiology, and are results of certain information stimulation in the social environment [22]. Long-term interaction between individual and a certain environment will form a corresponding lifestyle and physiological and psychological foundation. Therefore, the longer the drug addiction lasts, the longer the anti-drug treatment takes.

## 5. Limitations

A final note: firstly, although this study adopted the cohort study design, randomized grouping for subjects was not used, so there may be a selection bias. Secondly, since this study followed the strict inclusion and exclusion criteria and under rigorous experimental management, there may be some differences between the results of this clinical data and the real social data. Thirdly, only Shanghai was selected as a sample city to conduct the survey in this research program, which does not represent the whole situation of China. As this study was restricted to Shanghai only, it may not apply to international cohorts either, given the specificities of the organization system and the implementation process of treatment. Fourth, there are other factors associated with relapse which were not investigated, such as the convenience of obtaining drugs, social networks, drug use model, family environment, and social tolerance for the drug, and these factors are worth further study [23].

## 6. Conclusions

There are three factors influencing relapse based on the study, which included the years of drug use (OR = 1.078, *p* = 0.001), the compliance of participating in psychological counseling (OR = 3.563, *p* = 0.000) and the intervention model. That is, keeping the levels of other factors fixed, a one-year increase in the years of drug use results in a 0.078 unit increase in the relapse risk, one-level increase in the compliance of participating in psychological counseling results in a 2.563 times increase in the relapse risk, and the relapse risk of group 4 is 1.661 times higher than group 1. Therefore, appropriate and standard psychological counseling is very important for drug treatment. The longer the drug addiction lasts, the longer the anti-drug treatment takes.
